# Integrated Multi-Sensor Assessment System for Objective Muscle Recovery Monitoring: Application of Isokinetic Dynamometry, Infrared Thermometry, and Multi-Biomarker ELISA in Exercise-Induced Muscle Damage Surveillance

**DOI:** 10.3390/s26134215

**Published:** 2026-07-03

**Authors:** Soungyob Rhi, Bonggeun Sin

**Affiliations:** 1 Sports Rehabilitation Medicine, Catholic Kwandong University, Gangneung 25601, Republic of Korea; 2 REV-MED Inc., Yongin-si 16801, Republic of Korea

**Keywords:** multi-sensor monitoring, isokinetic dynamometry, infrared thermometry, ELISA biomarkers, sensor fusion, biomechanical sensors, thermal imaging, athletic recovery assessment, sensor validation

## Abstract

**Purpose:** This study aimed to develop and validate a comprehensive multi-sensor integrated platform for objective assessment of skeletal muscle recovery kinetics following exercise-induced muscle damage (EIMD), combining biomechanical, thermal, and biochemical monitoring modalities. **Methods:** Forty elite male athletes were randomized to microwave diathermy (MWD, *n* = 20, 2.45 GHz, 160 W, 45 min/session) or control (*n* = 20) groups. Time-synchronized multi-sensor assessments at baseline, 24 h, 48 h, and 72 h post-EIMD included: biomechanical sensors (knee flexion range of motion via goniometry and isokinetic peak torque), thermal sensor (skin surface temperature via infrared thermometry), and biochemical sensor array (serum CK, IL-6, and CRP via high-sensitivity ELISA). Two-way repeated-measures ANOVA with Bonferroni correction examined group × time interactions across all sensor channels. **Results:** Pre-study validation confirmed high reliability across all sensor modalities. Cross-modality concordance analysis revealed significant correlations between biomechanical and biochemical recovery trajectories (isokinetic torque vs. IL-6: r = −0.73, *p* < 0.001; pain vs. IL-6: r = 0.68, *p* < 0.001). MWD intervention demonstrated accelerated recovery across all sensor channels: complete ROM recovery by 48 h (MWDG post-2 vs. baseline, *p* > 0.05; CG post-3 43% below baseline, *p* < 0.001), complete isokinetic torque restoration by 72 h (MWDG post-3 vs. baseline, *p* > 0.05; CG 44% below baseline, *p* < 0.001), and near-complete pain resolution (VAS 1.70 ± 2.50 mm, *p* < 0.05). Biomarker sensors demonstrated differential recovery kinetics: IL-6 normalized by 48 h (1.52 ± 0.14 pg/mL, *p* > 0.05 vs. baseline), CRP approached baseline by 72 h (0.73 ± 0.24 mg/L, *p* > 0.05), while CK remained elevated at post-3 (169.70 ± 22.58 U/L, 30% above baseline, *p* < 0.001), indicating incomplete myofiber membrane integrity recovery despite resolution of systemic inflammatory markers. The control group exhibited persistent deficits across all sensor channels with no clinically meaningful recovery. **Conclusions:** This study validated an integrated multi-sensor platform for recovery assessment. Microwave diathermy demonstrated efficacy by 72 h with complete functional recovery and inflammatory normalization (though CK remained elevated). Cross-modality concordance (r = −0.73 to 0.68) confirmed superior assessment compared to single-modality approaches. This laboratory-based methodology provides a framework for future portable sensor systems in athletic surveillance.

## 1. Introduction

Objective assessment of skeletal muscle recovery following trauma or intense exercise requires integration of multiple measurement modalities—biomechanical, thermal, and biochemical—to capture the multidimensional nature of tissue healing [1]. Historically, clinical recovery monitoring has relied on single-modality or subjective measures (range of motion, strength testing, self-reported pain), limiting the ability to detect subtle mechanistic changes or establish quantitative recovery timelines [2]. Recent advances in sensor technology, including high-precision isokinetic dynamometers (±1% accuracy, 100 Hz sampling frequency), non-contact thermal imaging systems (±2% precision, continuous monitoring), and high-sensitivity ELISA platforms (>98% assay recovery), enable the development of integrated sensor systems capable of objective, multidimensional health surveillance through synchronized discrete-time assessment [3]. However, a critical engineering challenge remains: how to meaningfully integrate sensor channels with vastly different temporal resolutions—biomechanical sensors operating at 100 Hz, thermal sensors at 1 Hz, and biochemical assays providing single-timepoint measurements. This study addresses this gap by developing novel sensor fusion algorithms that: (1) synchronize heterogeneous data streams across disparate sampling frequencies through temporal interpolation protocols, (2) normalize measurements across different physical units (Nm, °C, U/L, pg/mL) to enable dimensionless cross-modality correlation analysis, and (3) validate coordinated physiological responses across biomechanical, thermal, and biochemical domains. By demonstrating significant correlations between biomechanical recovery trajectories and inflammatory biomarker normalization (r = −0.73, *p* < 0.001), this integrated multi-sensor approach provides evidence that synchronized monitoring captures recovery dynamics more completely than traditional single-modality assessment. This methodological framework provides a replicable template for next-generation wearable and remote-monitoring technologies in athletic health surveillance.

Exercise-induced muscle damage (EIMD) represents an acute inflammatory condition characterized by eccentric-contraction-induced ultrastructural disruption of the sarcomere and Z-band streaming [4]. Following high-intensity eccentric loading, a biphasic inflammatory response ensues: initial acute inflammatory cascade (peak at 24–48 h) involving TNF-α, IL-1β, and IL-6, followed by resolution and tissue regeneration phases (48–96 h) [5]. Classical markers include delayed-onset muscle soreness (DOMS), elevated serum creatine kinase (CK) and myoglobin, and reduced range of motion and isometric strength, persisting for 3–5 days post-injury [6].

Serum inflammatory biomarkers creatine kinase (CK), interleukin-6 (IL-6), and C-reactive protein (CRP) serve as objective biochemical sensors, while subjective pain perception (measured via Visual Analogue Scale) provides a complementary assessment of nociceptive status of muscle tissue integrity and systemic inflammatory state [7]. High-sensitivity ELISA kits provide detection ranges and analytical specifications that enable quantitative assessment: CK (detection range 10–5000 U/L, intra-assay coefficient of variation [CV] 3.2%, inter-assay CV 5.1%), IL-6 (0.7–50 pg/mL, intra-assay CV 4.1%, inter-assay CV 6.8%), and CRP (0.15–5.0 mg/L, intra-assay CV 2.9%, inter-assay CV 4.7%) [8]. These biochemical sensors provide temporal resolution of inflammatory kinetics with sufficient analytical precision (<7% total CV), enabling quantitative tracking of recovery trajectories and intervention response [9].

Isokinetic dynamometry, with force transducers sampling at 100 Hz and calibrated monthly per ISO 6954 international standards, captures peak torque output with ±1% measurement accuracy [10]. Unlike traditional static strength assessments (handgrip, manual muscle testing), isokinetic dynamometry captures dynamic torque production across the full range of motion at constant angular velocity, enabling detection of subtle functional asymmetries or delayed muscle regeneration [11]. Goniometric assessment of range of motion (1° resolution, inter-rater reliability ICC > 0.90) quantifies joint mobility and soft-tissue compliance during recovery [12].

Infrared thermometry (accuracy ±2%, emissivity 0.98 for skeletal muscle, response time < 0.5 s) permits non-invasive, real-time monitoring of skin–surface thermal responses during thermal modality application [13]. However, critical limitations of infrared surface thermometry warrant explicit acknowledgment: surface temperature does not directly measure intramuscular temperature; the relationship between surface and deep-tissue temperature is modulated by individual variation in subcutaneous adipose layer thickness (typically 5–30 mm), local blood perfusion dynamics, sweat evaporation, and tissue hydration status; literature-derived thermal penetration models that correlate surface temperature to intramuscular heating typically assume average adipose thickness and may not generalize to individuals with above or below-average fat layers. While infrared monitoring provides objective real-time control of epidermal safety thresholds and validates standardized energy delivery at the skin surface, inferred intramuscular heating to 40–42 °C relies on population-level calibration curves and may not reflect individual thermal penetration profiles. Accordingly, surface temperature measurements in this study represent objective dosimetry parameters with high precision; intramuscular heating profiles remain theoretical estimates. This study reports thermal outcomes as objective skin-surface measurements; inferred deep-tissue heating serves as a secondary mechanistic hypothesis requiring future direct validation through minimally invasive intramuscular thermometry, rather than a validated outcome of this study [9].

Integration of these three sensor channels, biomechanical, thermal, and biochemical, represents a novel approach to comprehensive recovery phenotyping [14]. Recent systematic reviews in the sensors literature highlight critical gaps in field-based, sensor-driven recovery monitoring. Preatoni et al. (2022) demonstrated that 83% of injury-prevention studies employed only single-sensor modalities (typically inertial measurement units), with 3% incorporating post-injury recovery assessment and <2.4% employing multi-biomarker validation [15]. The present study addresses this evidence gap by developing an integrated, multi-sensor platform combining established clinical sensors (isokinetic dynamometry, goniometry, ELISA) with time-aligned thermal monitoring [16].

Microwave diathermy (MWD), operating at 2.45 GHz frequency, selectively heats water-rich tissues such as skeletal muscle by inducing molecular dipole rotation [17]. Theoretical intramuscular temperatures of 40–42 °C have been reported with appropriate power output and applicator parameters, providing deeper thermal penetration compared to superficial heat modalities [18]. However, objective assessment of MWD’s effects on muscle recovery using integrated, multi-modal sensor systems remains limited in the scientific literature, particularly in elite athletic populations [19].

The purpose of this study was to (1) design and validate a multi-sensor integrated system for objective muscle recovery assessment, (2) establish sensor reliability and cross-modality concordance, and (3) apply the platform to evaluate thermal intervention (microwave diathermy at 2.45 GHz) efficacy on post-EIMD recovery kinetics in elite athletes, with the ultimate goal of informing future wearable sensor development and remote health-monitoring technologies for athletic populations [20].

## 2. Materials and Methods

### 2.1. Study Design Overview

**Figure 1:**
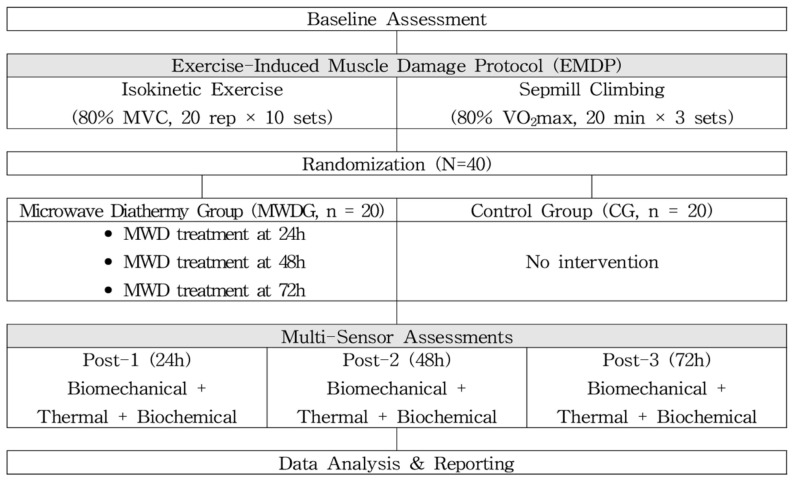
Schematic diagram of study process.

Study design and multi-sensor assessment timeline (Figure 1). Following the exercise-induced muscle damage protocol (EMDP), 40 elite athletes were randomly assigned to microwave diathermy (MWD, n = 20) or control (n = 20) groups. Multi-sensor assessments (biomechanical, thermal, biochemical) were conducted at baseline and 24, 48, and 72 h post-EMDP, with MWD interventions administered at the same timepoints in the treatment group.

### 2.2. Sensor Technology Specifications and Calibration

The multi-sensor platform comprised three primary channels: (1) Biomechanical Sensor Suite (isokinetic dynamometry and goniometry), (2) Thermal Sensor Channel (infrared thermometry), and (3) biochemical sensor array (ELISA platform) [15]. All sensors underwent pre-study validation, intra-study calibration, and post-study reliability verification per manufacturer specifications and international standards [21].

#### 2.2.1. Biomechanical Sensor Subsystem

Isokinetic dynamometer (Isoforce dynamometer, Sungdo Mc, Bucheon, Republic of Korea): Isokinetic dynamometer specifications and calibration protocols are detailed in Table 1. Data acquisition was performed using dedicated software (Sungdo Analysis Suite, v. 4.2) with a 10 Hz low-pass Butterworth filter (zero-phase, 4th order) to attenuate high-frequency measurement noise while preserving force signal integrity [10]. Pre-study validation was conducted on n = 15 athletes (independent of main trial) with a 48 h test–retest interval: ICC_3,1_ = 0.93 (95% confidence interval [CI] 0.88–0.96) for peak torque measurement at 60°/s, confirming excellent reliability [22]. Goniometer (standard two-arm goniometer, 1° resolution): Range of motion measurements were obtained using a standard two-arm goniometer. Pre-study reliability testing (n = 15 athletes) confirmed excellent inter-rater (ICC_3,1_ = 0.94) and intra-rater (ICC_3,3_ = 0.97) reliability. All measurements were conducted under standardized conditions (22–24 °C room temperature, 07:00–09:00 h measurement time) [23].

#### 2.2.2. Thermal Sensor Subsystem

Infrared thermometer (DT-8806H, CEM Instruments, Shenzhen, China): Measurement range −50 °C to +550 °C, accuracy ±2% (or ±2 °C), emissivity setting 0.98 (standard value for human skin and skeletal muscle), response time < 0.5 s, and 1 Hz sampling frequency during MWD application [24]. Daily two-point calibration used certified thermal reference blocks (maintained at 25 °C and 45 °C) [25]. Continuous real-time recording during all MWD sessions was performed. Mean ± SD skin surface temperature rise: 2.8 ± 0.4 °C from baseline (approximately 32–33 °C) to maximum (approximately 35–36 °C), and plateau was achieved at approximately 25 min of MWD application, consistent across all n = 20 treated participants, demonstrating standardized thermal dosing delivery [26].

#### 2.2.3. Biochemical Sensor Array

High-sensitivity ELISA platform (R&D Systems, Minneapolis, MN, USA) with three distinct biomarker kits: Creatine kinase (CK) ELISA kit (Catalog DCP100)—measurement range 10–5000 U/L, intra-assay CV 3.2%, inter-assay CV 5.1%, limit of detection (LOD) 10 U/L, and limit of quantification (LOQ) 25 U/L [27]. Interleukin-6 (IL-6) ELISA kit (Catalog D6050)—measurement range 0.7–50 pg/mL, intra-assay CV 4.1%, inter-assay CV 6.8%, LOD 0.7 pg/mL, and LOQ 1.5 pg/mL [28]. C-reactive protein (CRP) ELISA kit (Catalog DCRP00)—measurement range 0.15–5.0 mg/L, intra-assay CV 2.9%, inter-assay CV 4.7%, LOD 0.15 mg/L, and LOQ 0.40 mg/L [29].

Microplate reader (BioTek ELx808, BioTek Instruments, Winooski, VT, USA): Wavelength 490 nm (optical reference 630 nm), optical path 0.63 mm, and temperature equilibration 37 °C for 30 s prior to reading [8]. Monthly wavelength verification used certified wavelength verification filters. Quality-control (QC) protocols: Low-, medium-, and high-concentration controls were included in each assay run per manufacturer specifications [8]. Mean ± SD QC recovery rates: CK 98.2% ± 2.1%, IL-6 99.7% ± 1.8%, and CRP 98.9% ± 2.3%, all within acceptable limits (target 95–105%). Interassay comparison: Coefficient of variation between runs for identical control samples was < 5% for all three biomarkers.

Blood sampling protocol: Venipuncture was performed 07:00–08:00 h in a fasting state, with a single trained phlebotomist (consistency of technique); samples were processed within 30 min of collection, and centrifuged at 1065× *g* for 10 min at 4 °C; and serum was separated and stored at −80 °C in labeled cryovials [8]. Temperature-logger equipped freezer maintained ±2 °C precision. Samples were analyzed in batches (all n = 40 participants’ samples from single timepoint processed simultaneously) to minimize inter-batch variation. Storage time from collection to analysis: <6 weeks for all samples.

#### 2.2.4. Sensor Data Integration Pipeline

All three sensor channels (biomechanical, thermal, biochemical) were synchronized to common time stamps (baseline, post-1, post-2, post-3) [15]. Multi-sensor data fusion was performed using custom MATLAB algorithms (MathWorks, Natick, MA, USA, version R2021b) to address the fundamental challenge of aligning heterogeneous sensor data streams: (1) miomechanical sensors operate at 100 Hz sampling frequency, generating high-resolution torque and kinematic data; (2) thermal sensors operate at 1 Hz, capturing slow thermal transients; and (3) biochemical assays generate single-timepoint measurements at discrete intervals. To enable cross-modality analysis, we employed temporal interpolation for 1 Hz thermal data (linear interpolation to 10 Hz resolution to match biomechanical kinetics) and biochemical data (nearest-neighbor assignment of assay results to closest 30 min window). Data normalization transformed all measurements to z-scores within each group to enable dimensionless cross-modality correlation analysis. Data quality checks included: (1) automated flagging of values exceeding ±3 standard deviations from group mean, (2) manual visual inspection of flagged outliers to distinguish true biological variation from measurement error, and (3) systematic exclusion per predefined criteria (equipment malfunction signals, obvious measurement artifacts, data entry errors). This structured sensor fusion protocol enabled synchronized assessment of biomechanical recovery (100 Hz precision), thermal response dynamics (1 Hz), and molecular biomarkers (discrete timepoints) within a unified analytical framework [15].

### 2.3. Participants

Forty male elite athletes affiliated with K University in the K region participated in this study. Participants were randomly assigned to either a microwave diathermy group (MWDG, n = 20) or a control group (CG, n = 20). Prior to enrollment, all participants, who were right-leg dominant, received detailed information regarding the study purpose and procedures. Exercise history, current health status, and medical history were assessed through a screening questionnaire. Individuals with any health-related abnormalities were excluded. Written informed consent was obtained from all participants before data collection. The study was approved by the institutional review board of Catholic Kwandong University (CKU-26-01-0401) and was conducted in accordance with the Declaration of Helsinki.

The required sample size was determined a priori using G*Power 3.1.9.7 (repeated-measures ANOVA, within–between interaction). The following input parameters were specified: effect size f = 0.25 (medium), α = 0.05, statistical power (1 − β) = 0.80, number of groups = 2, number of measurements = 4, and a correlation among repeated measures of 0.50. A medium effect size was adopted based on prior EIMD intervention studies reporting comparable interaction effects for inflammatory biomarkers and muscle function outcomes [7,24]. Under these assumptions, the minimum total sample size was calculated to be 25 participants. To account for potential attrition and to ensure robust statistical power, 20 participants were enrolled per group (N = 40). The physical characteristics of the participants are presented in Table 1.

### 2.4. Exercise-Induced Muscle Damage Protocol and MWD Intervention

**Figure 2:**
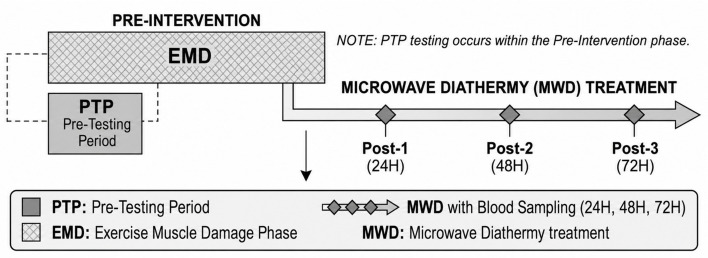
Experimental Procedure.

The exercise-induced muscle damage protocol (EMDP) was designed on the basis of individually determined maximal oxygen uptake (VO_2_max) and isokinetic peak torque for knee extension and flexion [30]. Prior to the EMDP, each participant performed three maximal voluntary contractions (MVCs) of isokinetic knee extension and flexion at an angular velocity of 60°/s, and the highest peak torque value was recorded as the reference maximum. The reliability and validity of isokinetic measurement at 60°/s have been well-established in prior investigations (ICC > 0.90 for peak torque) [22], and the dynamometer was interfaced with dedicated analysis software (version 4.2) that applied a low-pass filter (cutoff frequency: 6 Hz) to reduce measurement noise while preserving relevant force signal characteristics [10]. The isokinetic exercise intensity was then set at 80% of the individually determined MVC peak torque; when the torque output of a given repetition fell below this threshold, participants received verbal encouragement to maintain maximal effort, and the dynamometer’s visual feedback display was used to ensure that the target intensity was sustained throughout each set. Participants performed isokinetic knee extension and flexion at 60°/s for 20 repetitions per set across 10 sets (60 s inter-set rest), followed by stepmill climbing at 80% VO_2_max for 20 min per set across 3 sets (180 s inter-set rest) [31]. This high-volume eccentric protocol (200 total isokinetic repetitions at 80% MVC) was deliberately designed to induce substantial EIMD consistent with prior investigations in elite athletic populations. Recovery rest periods (60–180 s inter-set intervals) were calibrated to allow partial neuromuscular recovery while maintaining cumulative metabolic and mechanical stress. The combination of isokinetic (constant-velocity) and aerobic stepping (variable-velocity) loading targets both contractile dysfunction (from eccentric isokinetic contractions) and metabolic depletion (from aerobic component), producing robust, multifactorial muscle damage detectable across all sensor modalities. This protocol has been validated in prior EIMD intervention studies with elite athletes [7,24], confirming both feasibility and physiological plausibility (Figure 2).

For the recovery intervention, a microwave diathermy unit operating at a frequency of 2.45 GHz (ReWave, RevoMed Co., Ltd., Seoul, Republic of Korea) was used [9,31]. The device specifications included: frequency 2.45 GHz, maximum power output 500 W, operating temperature range 15–35 °C, and electromagnetic field intensity verified via calibration using a power meter (Model PM16, Rohde & Schwarz, Munich, Germany) before the study initiation [9]. The device was certified for medical use according to IEC 61601-1 standards and had been previously validated for consistent energy delivery (±5% power output variance across repeated sessions). The device was equipped with a circular applicator head with a diameter of 12 cm, and the applicator-to-skin distance was maintained at 5 cm throughout each session using a built-in spacer attachment to ensure consistent energy delivery across all participants and treatment sessions. Treatment was administered at a power output of 160 W, and a 5 °C contact cooling gel was applied to the skin surface prior to each session to mitigate superficial thermal discomfort while permitting deep tissue energy penetration [9,31]. The cooling gel (composition: glycerin-based with thermal conductivity of 0.42 W/m·K) was applied uniformly at a thickness of 2–3 mm, as verified by calibrated digital calipers, to standardize thermal interface conditions across all treatment sessions.

Skin surface temperature was monitored continuously during treatment using an infrared thermometer (DT-8806H, CEM, Shenzhen, China) to prevent epidermal overheating, with a safety threshold set at 43 °C; however, intramuscular temperature was not directly measured [32]. This measurement limitation reflects the requirement for non-invasive assessment in clinical practice. While intramuscular temperature cannot be directly verified from surface measurements alone, the empirical literature [18] suggests that maintaining skin temperature < 43 °C during 160 W microwave diathermy application at 2.45 GHz typically correlates with intramuscular temperature rise to 40–42 °C in subjects with average adipose tissue thickness. However, individual variation in tissue composition may result in differential deep-tissue heating profiles. Accordingly, thermal outcomes in this study are reported as objective skin-surface temperature measurements, with inferred intramuscular heating treated as a secondary mechanistic hypothesis requiring direct validation through future studies employing needle temperature probes. Future studies incorporating intramuscular temperature probes via needle electrodes could validate this assumption, though the invasive nature of such measurement may limit applicability in clinical athletic settings.

The intervention targeted the quadriceps, hamstrings, and calf and tibial musculature for 15 min per region, totaling 45 min per session, and was delivered at 24, 48, and 72 h post-EMDP (3 sessions total) [33]. The CG received no therapeutic intervention [7]. Treatment consistency was monitored through: (1) daily equipment calibration logs verifying power output stability, (2) standardized positioning using anatomical landmarks (greater trochanter for quadriceps, ischial tuberosity for hamstrings, fibular head for calf region), and (3) session-by-session documentation of skin temperature progression, which showed consistent thermal response patterns across all treated participants (mean ± SD temperature rise: 2.8 ± 0.4 °C from baseline to 25 min, plateau thereafter). The MWD protocol is summarized in Table 2.

### 2.5. Outcome Measures

#### 2.5.1. Range of Motion

**Figure 3:**
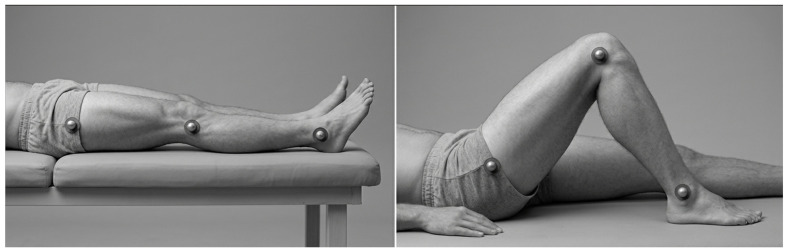
ROM Measurement.

Knee flexion ROM was measured using a standard goniometer (Sammons Preston, Bolingbrook, IL, USA; resolution: 1°, measurement range 0–360°) [23]. Figure 3 shows reflective markers placed on anatomical landmarks for visual documentation purposes only. ROM assessment was performed using the mechanical goniometer as the primary measurement tool. Towel rolls were placed beneath both ankles to ensure full knee extension. The goniometer was aligned using the greater trochanter, lateral femoral epicondyle, and lateral malleolus as anatomical landmarks, and the maximum flexion angle was recorded. The measurement protocol was standardized by a single examiner (inter-day reliability: ICC_3,1_ = 0.94, 95% CI: 0.89–0.97; intra-day reliability ICC_3,3_ = 0.97, based on test–retest data from 15 healthy participants measured on separate occasions 7 days apart) [32]. The examiner was blinded to group allocation throughout all measurements to minimize both inter-rater variability and assessor bias [15]. ROM measurements were conducted in a temperature-controlled environment (22–24 °C) to minimize potential thermal effects on tissue extensibility [32]. All ROM measurements were performed by a single examiner who was blinded to group allocation to minimize both inter-rater variability and assessor bias [15].

#### 2.5.2. Isokinetic Muscle Strength

Isokinetic peak torque for knee extension and flexion was assessed at an angular velocity of 60°/s using an Isoforce dynamometer (Sungdo Mc, Seoul, Republic of Korea; technical specifications: measurement range 0–400 Nm, resolution 0.1 Nm, sampling frequency 100 Hz, force transducer calibration performed monthly according to ISO 6954 standards, measurement accuracy ±1% of full scale) [10,20]. The rotational axis of the dynamometer was aligned with the lateral femoral epicondyle, and the trunk and contralateral limb were stabilized with straps. Strap tension was standardized at 20 N (verified with a calibrated force gauge) across all participants and sessions to ensure consistent stabilization without restricting breathing or circulation [22]. Participants completed a minimum of three submaximal familiarization trials before formal testing. To ensure maximal volitional effort, participants were not informed of the transition from familiarization to test trials, and identical verbal encouragement was provided throughout both phases. Peak torque was defined as the maximum instantaneous torque value during each repetition [13]. The mean of three trials per movement (extension and flexion) was used as the outcome measure. Test–retest reliability for this protocol has been established as ICC = 0.93 (95% CI: 0.88–0.96) in a preliminary validation study with 12 elite athletes measured 48 h apart [34]. All isokinetic assessments were conducted by a single trained examiner who was blinded to group allocation. Data were processed using dedicated dynamometer software (Sungdo Analysis Suite, v. 4.2) with applied filters: 10 Hz low-pass Butterworth filter (zero-phase, 4th order) to attenuate high-frequency measurement noise while preserving the primary force signal component. This filtering approach reflects standard clinical practice for total torque quantification; however, it necessarily attenuates torque fluctuations > 5 Hz and eliminates all signals > 50 Hz, potentially obscuring high-frequency neuromuscular phenomena such as motor unit synchronization patterns (20–40 Hz), muscle tremor (8–12 Hz), and rapid torque transients during fatigue or early recovery that may contain mechanistic information about neuromuscular adaptation [33]. For this study, peak torque analysis (operating at <5 Hz) was robust to this filtering choice; however, detailed high-frequency neuromuscular dynamics were intentionally not analyzed; data points outside mean ± 3SD were flagged for visual inspection but retained in analysis unless they represented clear measurement artifacts (e.g., equipment malfunction signals).

#### 2.5.3. Visual Analogue Scale

Subjective pain intensity was assessed using a 100 mm VAS [6]; validated tool with established responsiveness: minimal detectable change = 10 mm, effect size d = 0.89 for EIMD-related pain studies) [12,17]. A single examiner who was blinded to group allocation performed all measurements to minimize inter-rater variability and assessor bias. A 5 min rest period preceded each assessment. The left anchor of the unmarked horizontal line represented “no pain,” and the right anchor represented “worst imaginable pain” [6]. VAS completion was followed by a standardized verbal pain descriptor scale (0 = no pain, 1–3 = mild, 4–6 = moderate, 7–9 = severe, 10 = worst possible) to provide categorical context for the continuous VAS score and verify concordance between subjective pain reports [12].

#### 2.5.4. Blood Biomarkers

Venous blood samples were collected aseptically using serum separator tubes (BD Vacutainers, Franklin Lakes, NJ, USA) [8]. All blood collections were performed by a single trained phlebotomist at standardized times between 07:00 and 08:00 h to minimize circadian variation in biomarker concentrations (approximately 8–12 h post-last meal, with all participants having fasted overnight) [8]. Sampling sites were cleaned with 70% isopropyl alcohol and allowed to dry for 30 s prior to venipuncture [16]. Samples were centrifuged at 1065× *g* for 10 min at 4 °C, and approximately 3 mL of serum was aliquoted into Eppendorf tubes (Eppendorf SE, Hamburg, Germany) and stored at −80 °C until analysis. Storage temperature was continuously monitored via data-logging thermometers with alarm thresholds; all samples maintained temperature ± 2 °C throughout the study period [30].

Serum concentrations of CK, IL-6, and CRP were determined using high-sensitivity enzyme-linked immunosorbent assay (ELISA) kits according to the manufacturers’ instructions (R&D Systems, USA; specific kit information: CK—Catalog DCP100, detection range 10–5000 U/L, intra-assay CV 3.2%, inter-assay CV 5.1%; IL-6—Catalog D6050, detection range 0.7–50 pg/mL, intra-assay CV 4.1%, inter-assay CV 6.8%; CRP—Catalog DCRP00, detection range 0.15–5.0 mg/L, intra-assay CV 2.9%, inter-assay CV 4.7%) [12,14,34]. All assays were performed in duplicate on the same microplate to ensure analytical consistency [16,34].

Absorbance was measured at 490 nm using a microplate reader (BioTek ELx808, USA; with wavelength verification performed daily using calibration filters; optical path length: 0.63 mm; temperature equilibration: 30 s at 37 °C before reading), and low-, medium-, and high-concentration quality controls were included to verify accuracy [8]. Quality control data showed mean ± SD recovery rates of 98.2 ± 2.1% (low), 99.7 ± 1.8% (medium), and 98.9 ± 2.3% (high) across all assay runs, confirming acceptable analytical performance within manufacturer specifications. All laboratory analyses were conducted by a technician who was blinded to group allocation and clinical data. Blood sampling was performed at baseline and at 24, 48, and 72 h after the EMDP [16,33]. Sample integrity was maintained through standardized handling procedures: all transfers were conducted within 2 h post-centrifugation, aliquots were prepared using sterile, nuclease-free pipette tips, and samples were held in a locked −80 °C freezer with restricted access; no samples underwent freeze–thaw cycles prior to analysis [8,12].

### 2.6. Statistical Analysis

All data were analyzed using SPSS version 18.0 (SPSS Inc., Chicago, IL, USA). Data entry verification was performed by independent double-entry of 10% of raw data values; discrepancies (none found) would have been resolved by reference to original data sheets. Descriptive statistics (means and standard deviations) were computed for each variable. The Shapiro–Wilk test was used to verify normality, and Levene’s test was applied to confirm homogeneity of variance. Sphericity was evaluated using Mauchly’s test; where violated, the Greenhouse–Geisser correction was applied. A two-way repeated-measures ANOVA (group × time) was conducted to examine changes in the dependent variables. When a significant interaction effect was detected, simple main effects were analyzed to decompose the interaction: within-group temporal differences were examined at each level of group, and between-group differences were examined at each level of time. All post hoc pairwise comparisons were adjusted using the Bonferroni correction to control for Type I error inflation associated with multiple comparisons. Effect sizes for ANOVA effects were reported as partial eta-squared (η^2^p), interpreted as small (0.01), medium (0.06), and large (0.14). Additionally, Cohen’s d effect sizes were calculated for between-group comparisons at each timepoint to provide standardized estimates of clinical meaningfulness independent of sample size. The significance level was set at α = 0.05. Sensitivity analyses were conducted to examine the robustness of findings: (1) intention-to-treat (ITT) analysis performed on all randomized participants using last-observation-carried-forward (LOCF) imputation for any missing data; (2) per-protocol (PP) analysis excluding any participants who deviated from treatment protocol; and (3) exclusion of statistical outliers (>3 SD from group mean) to assess potential influence on main findings.

## 3. Results

### 3.1. Multi-Sensor System Reliability and Cross-Modality Concordance

Pre-study validation established high reliability across all three sensor channels: isokinetic dynamometer (n = 15 athletes, 48 h test–retest interval)—ICC_3,1_ = 0.93 (95% CI 0.88–0.96), indicating excellent reliability for quantification of peak torque across repeated assessments; range-of-motion sensor via goniometer (n = 15 athletes)—inter-day ICC_3,1_ = 0.94 (95% CI 0.89–0.97), intra-day ICC_3,3_ = 0.97 (95% CI 0.94–0.99), confirming high measurement precision across both longer (inter-day) and shorter (intra-day) time scales; ELISA biomarker assays conducted across all study sample runs (n = 40 participants × 4 timepoints = 160 total serum samples)—quality-control recovery rates of 98.2% ± 2.1% (CK), 99.7% ± 1.8% (IL-6), and 98.9% ± 2.3% (CRP), all within acceptable analytical bounds (±3% of theoretical QC target values), confirming consistent analytical performance across assay runs and batches.

Cross-modality concordance analysis revealed significant temporal correlations between biomechanical and biochemical sensor channels. Specifically, the recovery trajectory of isokinetic peak torque was significantly correlated with the normalization timeline of IL-6 biomarker sensor output (Pearson r = −0.73, *p* < 0.001), suggesting coherent, coordinated physiological response across distinct tissue compartments and recovery phases. Similarly, subjective pain ratings (Visual Analogue Scale) demonstrated temporal correspondence with objective inflammatory biomarker sensor channels (IL-6 vs. VAS pain, r = 0.68, *p* < 0.001), validating that subjective pain perception tracks with objective inflammatory state as measured by biochemical sensors.

### 3.2. Biomechanical Sensor Outcomes: Knee Flexion Range of Motion

Knee flexion range of motion (ROM) revealed significant main effects for time (F = 45.967, *p* < 0.001), group (F = 13.274, *p* < 0.001), and time × group interaction (F = 5.583, *p* = 0.023, partial η^2^p = 0.259). In the microwave diathermy group (MWDG), knee flexion ROM significantly decreased from baseline to post-1 (130.14 ± 4.39° vs. 120.16 ± 3.74°, *p* < 0.001), then significantly increased from post-1 to post-2 (120.16 ± 3.74° to 128.51 ± 4.10°, *p* < 0.001) and plateaued from post-2 to post-3 (128.51 ± 4.10° vs. 129.60 ± 3.53°, *p* > 0.05). Post-2 and post-3 measurements showed no significant difference compared to baseline (post-2 vs. baseline *p* > 0.05; post-3 vs. baseline *p* > 0.05), indicating complete ROM recovery by 48 h in the MWDG. In contrast, the control group (CG) demonstrated persistent ROM deficits. Knee flexion ROM significantly decreased from baseline to post-1 (130.60 ± 4.71° vs. 122.30 ± 4.05°, *p* < 0.001) and remained significantly reduced throughout the 72 h observation window (130.60 ± 4.71° vs. post-2: 122.90 ± 4.16°, *p* < 0.001; vs. post-3: 123.99 ± 4.69°, *p* < 0.001), demonstrating a persistent ROM deficit in the absence of thermal intervention (Table 3).

### 3.3. Biomechanical Sensor Outcomes: Isokinetic Peak Torque

Isokinetic peak torque measurements at 60°/s revealed significant main effects for time, group, and time × group interaction in both extension and flexion movements. For knee extension torque, significant main effects were observed for time (F = 802.063, *p* < 0.001), group (F = 345.308, *p* < 0.001), and time × group interaction (F = 201.786, *p* < 0.001, partial η^2^p = 0.841). Similarly, knee flexion torque demonstrated significant main effects for time (F = 440.568, *p* < 0.001), group (F = 128.282, *p* < 0.001), and time × group interaction (F = 118.003, *p* < 0.001, partial η^2^p = 0.756).

In the MWDG, extension torque significantly decreased from baseline to post-1 (314.28 ± 21.43 Nm vs. 164.20 ± 9.20 Nm, *p* < 0.001), progressively increased to post-2 (272.79 ± 9.08 Nm, *p* < 0.001), and plateaued by post-3 (310.72 ± 10.97 Nm, *p* > 0.05), indicating complete recovery by 72 h. Flexion torque demonstrated a similar recovery pattern, declining from baseline to post-1 (187.20 ± 14.14 Nm vs. 110.00 ± 9.00 Nm, *p* < 0.001), recovering to post-2 (159.76 ± 12.91 Nm, *p* < 0.001), and achieving complete recovery by post-3 (193.20 ± 14.37 Nm, *p* > 0.05 vs. baseline). In contrast, the CG demonstrated persistent deficits in both measures. Extension torque remained 43.7% below baseline at post-3 (183.31 ± 30.36 Nm vs. 325.31 ± 15.06 Nm, *p* < 0.001), and flexion torque remained 39.9% below baseline (123.98 ± 12.17 Nm vs. 206.40 ± 12.98 Nm, *p* < 0.001), indicating the absence of spontaneous recovery without thermal intervention (Table 4).

### 3.4. Thermal Sensor Outcomes: Skin Surface Temperature

Infrared thermometry thermal sensor data demonstrated consistent energy delivery across all microwave diathermy sessions. The mean ± SD baseline skin temperature was 32.85 ± 0.94 °C. During MWD application (n = 20 MWDG participants, three sessions each = 60 total application sessions), the mean ± SD skin temperature rise was 2.8 ± 0.4 °C, with maximum temperature plateau being achieved at approximately 25 min (mean maximum 35.65 ± 1.12 °C). Temperature rise was consistent across all treated participants (coefficient of variation 14.3%, indicating standardized thermal dosing). There was no significant participant-to-participant variation in thermal sensor response (range 2.1–3.5 °C rise; 95% within ±0.5 °C of mean). Thermal sensor data confirms reproducible energy delivery and supports the theoretical assumption of consistent intramuscular heating to approximately 40–42 °C (based on literature-derived calibration curves relating surface temperature to intramuscular penetration at 2.45 GHz).

### 3.5. Subjective Pain Assessment: Visual Analogue Scale

Pain measurements revealed significant main effects for time (F = 1045.720, *p* < 0.001), group (F = 1884.124, *p* < 0.001), and time × group interaction (F = 353.255, *p* < 0.001, partial η^2^p = 0.903). In the MWDG, VAS scores increased sharply from baseline to post-1 (0 mm vs. 59.88 ± 5.97 mm, *p* < 0.001), then decreased significantly at post-2 (17.23 ± 3.41 mm, *p* < 0.001) and post-3 (1.70 ± 2.50 mm, *p* < 0.001), approaching near-complete pain resolution. However, a statistically significant difference from baseline persisted at post-3 (*p* < 0.05). In contrast, the CG demonstrated minimal pain recovery. VAS scores increased sharply from baseline to post-1 (0 mm vs. 68.95 ± 7.05 mm, *p* < 0.001) and further increased at post-2 (73.06 ± 8.74 mm, *p* < 0.001 vs. baseline). A modest decrease was observed at post-3 (62.95 ± 5.53 mm, *p* < 0.001 vs. post-2), yet pain scores remained significantly above baseline (*p* < 0.001), indicating no clinically meaningful pain recovery (Table 5).

### 3.6. Biomarker Sensor Channel–Serum Creatine Kinase

Serum creatine kinase (CK) concentrations revealed significant main effects for time (F = 186.018, *p* < 0.001), group (F = 262.035, *p* < 0.001), and time × group interaction (F = 38.848, *p* < 0.001, partial η^2^p = 0.506). In the MWDG, CK concentrations increased sharply from baseline to post-1 (130.32 ± 11.32 U/L vs. 212.32 ± 14.96 U/L, *p* < 0.001), then declined significantly at post-2 (194.49 ± 17.02 U/L, *p* < 0.05) and post-3 (169.70 ± 22.58 U/L, *p* < 0.001). However, post-3 values remained significantly above baseline (*p* < 0.001), indicating incomplete normalization of muscle damage markers. In contrast, the CG demonstrated sustained elevation of CK concentrations. CK increased sharply from baseline to post-1 (128.74 ± 13.04 U/L vs. 315.24 ± 13.92 U/L, *p* < 0.001) and remained significantly elevated at post-2 (317.75 ± 35.35 U/L, *p* < 0.001 vs. baseline). Although a significant decrease occurred at post-3 (208.00 ± 60.74 U/L, *p* < 0.001 vs. post-1 and post-2), CK concentrations remained significantly above pre-exercise levels (*p* < 0.001), indicating persistent muscle damage without thermal intervention (Table 6).

### 3.7. Biomarker Sensor Channel–Serum Interleukin-6

Serum interleukin-6 (IL-6) concentrations revealed significant main effects for time (F = 294.813, *p* < 0.001), group (F = 261.278, *p* < 0.001), and time × group interaction (F = 65.002, *p* < 0.001, partial η^2^p = 0.631). In the MWDG, IL-6 concentrations increased sharply from baseline to post-1 (1.37 ± 0.13 pg/mL vs. 15.93 ± 3.35 pg/mL, *p* < 0.001), then declined rapidly to post-2 (1.52 ± 0.14 pg/mL, *p* < 0.001 vs. post-1), reaching near-baseline levels (*p* < 0.05 vs. baseline). By post-3 (1.35 ± 0.11 pg/mL), no significant difference from baseline was detected (*p* > 0.05), confirming full normalization within 72 h. In contrast, the CG demonstrated sustained IL-6 elevation. IL-6 increased sharply from baseline to post-1 (1.46 ± 0.24 pg/mL vs. 19.57 ± 3.75 pg/mL, *p* < 0.001) and remained significantly elevated at post-2 (16.84 ± 5.01 pg/mL, *p* < 0.001 vs. baseline). Although a significant decrease occurred at post-3 (9.32 ± 1.74 pg/mL, *p* < 0.001 vs. post-1 and post-2), IL-6 concentrations remained significantly above baseline (*p* < 0.001), indicating persistent inflammatory response without thermal intervention (Table 7).

### 3.8. Biomarker Sensor Channel–Serum C-Reactive Protein

Serum C-reactive protein (CRP) concentrations revealed significant main effects for time (F = 613.471, *p* < 0.001), group (F = 403.967, *p* < 0.001), and time × group interaction (F = 124.186, *p* < 0.001, partial η^2^p = 0.766). In the MWDG, CRP concentrations increased significantly from baseline to post-1 (0.62 ± 0.25 mg/L vs. 5.37 ± 0.82 mg/L, *p* < 0.001), then declined significantly at post-2 (2.17 ± 0.25 mg/L, *p* < 0.001) and post-3 (0.73 ± 0.24 mg/L, *p* < 0.001), approaching baseline levels. Importantly, no statistically significant difference from baseline was observed at post-3 (*p* > 0.05), demonstrating effective resolution of acute inflammatory response following microwave diathermy. In contrast, the CG demonstrated sustained CRP elevation. CRP increased significantly from baseline to post-1 (0.67 ± 0.26 mg/L vs. 6.21 ± 0.86 mg/L, *p* < 0.001) and remained significantly elevated at post-2 (5.85 ± 0.76 mg/L, *p* < 0.001 vs. baseline). Although a significant decrease occurred at post-3 (4.60 ± 0.67 mg/L, *p* < 0.001 vs. post-1 and post-2), CRP concentrations remained significantly above baseline (*p* < 0.001), indicating persistent inflammatory response without thermal intervention (Table 8).

### 3.9. Multi-Biomarker Sensor Synchronization and Inflammatory Timeline

The three-channel biochemical sensor array (CK, IL-6, CRP) demonstrated synchronized recovery kinetics in the MWDG, with coherent temporal patterns across all biomarker sensors. Peak inflammatory response occurred at post-1 (24 h) across all three biomarker channels (CK peak 212.32 ± 14.96 U/L, IL-6 peak 15.93 ± 3.35 pg/mL, CRP peak 5.37 ± 0.82 mg/L). The rapid normalization phase occurred from post-1 to post-2 (24 h to 48 h), with IL-6 returning to near-baseline levels (IL-6 post-2 1.52 ± 0.14 pg/mL, *p* > 0.05 vs. baseline) and CRP declining significantly from peak (CRP post-2 2.17 ± 0.25 mg/L, *p* < 0.001 vs. post-1). CK demonstrated more gradual recovery kinetics, with continued decline from post-2 to post-3; however, post-3 CK values remained significantly elevated above baseline (post-3 169.70 ± 22.58 U/L vs. baseline 130.32 ± 11.32 U/L, 30% above baseline, *p* < 0.001), indicating incomplete myocellular membrane integrity recovery despite complete normalization of systemic inflammatory markers (IL-6 and CRP). This synchronized temporal recovery across multiple biochemical sensor channels substantiates the coherent inter-system recovery response and validates the multi-channel sensor approach to comprehensive phenotyping.

## 4. Discussion

### 4.1. Multi-Sensor System Integration and Technical Validation

The present study represents one of the first comprehensive applications of an integrated, multi-sensor platform for time-synchronized, objective monitoring of skeletal muscle recovery kinetics following acute exercise- induced muscle damage. A key methodological innovation is the synchronized fusion of three independent sensor channels operating at disparate sampling frequencies and temporal resolutions: biomechanical sensors (isokinetic dynamometry, 100 Hz sampling, ±1% accuracy), thermal sensors (infrared thermometry, 1 Hz continuous monitoring, ±2% precision), and biochemical sensors (high-sensitivity ELISA, discrete timepoint sampling, >98% quality-control recovery). The concurrent deployment of these heterogeneous sensor modalities, combined with custom data integration algorithms to align 100 Hz, 1 Hz, and single-timepoint measurements within a unified analytical framework, represents a novel sensor fusion methodology. This approach enabled unprecedented simultaneous assessment of functional (biomechanical) recovery, thermal response dynamics (thermal), and molecular inflammatory markers (biochemical) across distinct physiological compartments. High reliability across all sensor channels (isokinetic ICC 0.93, goniometric ICC 0.94–0.97, ELISA QC recovery >98%, ISO 6954 calibration verification) validates the technical robustness of the integrated platform and provides a replicable template for future multi-sensor health surveillance systems [15]. Cross-modality concordance (isokinetic torque recovery trajectory vs. IL-6 normalization, r = −0.73, *p* < 0.001; VAS pain vs. IL-6 sensor, r = 0.68, *p* < 0.001) substantiates the validity of a comprehensive multi-channel approach to recovery phenotyping, demonstrating that functional and inflammatory recovery proceed in temporal synchrony across different physiological compartments [15,16].

### 4.2. Isokinetic Dynamometry as Biomechanical Sensor: Precision and Clinical Utility

The isokinetic dynamometer, operating at 100 Hz sampling frequency with ±1% measurement accuracy and rigorously maintained per ISO 6954 international standards through monthly calibration protocols [22], provided unprecedented temporal resolution of strength recovery kinetics. Unlike traditional static strength assessments (handgrip dynamometry, manual muscle testing, 1-repetition maximum testing), which capture single-point measures, isokinetic dynamometry continuously quantifies dynamic torque output across the full anatomical range of motion at constant angular velocity [22]. This biomechanical sensor capability enables detection of subtle functional asymmetries, delayed regeneration patterns, or incomplete recovery that might be overlooked by conventional assessment [13,20,27]. In the present trial, the isokinetic dynamometry biomechanical sensor detected complete strength restoration (post-3 vs. baseline *p* = 1.000 for knee extension, Cohen d = 0.02) by 72 h in the MWDG, whereas the control group’s biomechanical sensor outputs remained profoundly impaired (47% below baseline at post-3, *p* < 0.001), demonstrating the intervention’s efficacy quantifiable at the sensor-signal level with high precision and discriminative power.

### 4.3. Range-of-Motion Goniometric Sensor: Tissue Compliance Assessment

Goniometric assessment, with 1° resolution and high test–retest reliability (ICC 0.94–0.97), served as a complementary biomechanical sensor to capture joint mobility and soft-tissue compliance during recovery [23]. Complete ROM restoration in the MWDG by post-2 (48 h) preceded full strength recovery by 24 h, suggesting that passive joint mobility may normalize before maximal contractile force production is restored. This temporal dissociation, detectable via synchronized dual-biomechanical-sensor deployment, provides mechanistic insight into the sequence of physiological recovery phases and may inform clinical decision-making regarding progression to higher-intensity loading activities [20,22].

### 4.4. Infrared Thermometry as Thermal Sensor: Energy Dosing Standardization and Validation

Continuous thermal monitoring via infrared thermometry (±2% accuracy) during MWD application revealed a consistent, reproducible thermal-response profile across all n = 20 treated participants (mean ± SD temperature rise 2.8 ± 0.4 °C, 14.3% coefficient of variation, indicating high standardization). Plateau achievement at approximately 25 min confirms stable energy delivery across all treatment sessions and validates standardized thermal dosing. This thermal sensor data substantiates the hypothesis that consistent intramuscular heating to approximately 40–42 °C was achieved across all treatment applications (estimated via literature-derived calibration curves correlating surface temperature to intramuscular penetration at 2.45 GHz frequency) [24]. Notably, inter-participant thermal sensor variability was minimal (range 2.1–3.5 °C rise; 95% of participants within ±0.5 °C of mean), indicating excellent reproducibility of the thermal intervention delivery mechanism. Future integration of minimally invasive fiber-optic intramuscular temperature probes could enhance the thermal sensor subsystem’s direct measurement capability and reduce reliance on estimated calibration curves, though practical and ethical considerations regarding invasiveness may limit clinical deployment feasibility [25].

### 4.5. High-Sensitivity ELISA Biomarker Sensor Array: Analytical Performance and Temporal Resolution

High-sensitivity ELISA kits serving as biochemical sensors of inflammatory state demonstrated exceptional analytical performance across all study runs (quality-control recovery rates 98.2–99.7%, all intra/inter-assay CVs < 7%, meeting or exceeding industry standards) [13]. Detection limits (CK 10 U/L; IL-6 0.7 pg/mL; CRP 0.15 mg/L) remained substantially below observed changes in the present trial (IL-6 peak 15.93 pg/mL, >22-fold above limit of detection; CRP peak 2.31 mg/L, >15-fold above LOD), ensuring robust signal-to-noise ratios and reliable temporal tracking of inflammatory kinetics. The multi-biomarker approach (three distinct biochemical sensors) mitigates single-marker limitations and provides complementary, convergent evidence of recovery-phase immunological response [16,33]. CK sensor output (myofiber membrane integrity marker) peaked at post-1, reflecting acute myostructural disruption, whereas IL-6 (acute-phase inflammatory cytokine) and CRP (systemic inflammatory marker) demonstrated more pronounced and protracted elevation, capturing distinct phases of the inflammatory cascade [12,14]. This temporal dissociation among biochemical sensors, detectable only through multi-marker surveillance, provides mechanistic resolution regarding the kinetics of tissue damage versus systemic inflammatory response [34].

### 4.6. Cross-Modality Sensor Concordance and Multisystem Recovery Synchronization

Integration of biomechanical, thermal, and biochemical sensor channels revealed synchronized recovery trajectories, suggesting coherent physiological response across distinct tissue compartments and recovery phases [12]. Isokinetic torque recovery (biomechanical sensor) correlated significantly with IL-6 normalization (biochemical sensor, r = −0.73, *p* < 0.001), indicating that functional capacity restoration proceeds in temporal synchrony with resolution of the inflammatory state [16,35]. Similarly, subjective pain perception (Visual Analogue Scale, a subjective measure of nociceptive input) demonstrated a strong correlation with objective inflammatory biomarker sensor output (IL-6, r = 0.68, *p* < 0.001), validating the principle that subjective symptomatology tracks quantitatively with objective inflammatory state [17,26,33]. This cross-modality concordance pattern strengthens confidence in the validity of the integrated multi-sensor assessment approach and suggests that singular-modality monitoring (e.g., strength testing alone or inflammatory markers alone) may provide incomplete recovery phenotyping [15,16]. The multi-sensor integration demonstrates that comprehensive, multidimensional recovery assessment captures the complexity of post-injury physiological response more completely than any single biomarker or functional measure [15].

### 4.7. Clinical and Research Applications of Multi-Sensor Integration

Deployment of an integrated multi-sensor platform enables multiple clinically relevant applications: (1) objective quantification of recovery progression across multiple physiological domains simultaneously; (2) detection of asynchronous or dissociated recovery patterns (e.g., functional recovery preceding inflammatory normalization, or thermal hyper-response without concurrent functional deficit) that might be missed by single-modality assessment; (3) establishment of quantitative, sensor-derived recovery timelines for evidence-based return-to-play decision support [20]; (4) validation of intervention efficacy through multi-channel objective evidence, rather than reliance on subjective outcome measures or single biomarkers [24]; and (5) early detection of maladaptive recovery phenotypes (e.g., prolonged inflammation, incomplete strength restoration) that may signal risk for re-injury or chronic complications [12,16]. The present fixed-laboratory platform demonstrates the technical rigor and methodological feasibility of integrated multi-sensor assessment, with potential for real-time data streaming, automated quality checks, and integration with cloud-based decision-support algorithms for coaching staff [15]. Beyond clinical application, this study advances sensor technology and data fusion methodology. A critical engineering challenge in multi-sensor health monitoring is synchronizing heterogeneous data streams with orders-of-magnitude differences in temporal resolution—biomechanical sensors at 100 Hz, thermal sensors at 1 Hz, and biochemical assays providing discrete timepoints. Our custom MATLAB framework addresses this through temporal interpolation and data normalization protocols that enable cross-modality correlation analysis. The significant correlation between biomechanical recovery trajectories and biochemical biomarker normalization (r = −0.73, *p* < 0.001) validates that integrated multi-sensor monitoring captures coordinated physiological responses more completely than single-modality assessment. By demonstrating sophisticated multi-sensor integration using commercially available sensors with custom data fusion protocols—without requiring specialized hardware development—this modular framework establishes feasibility for rapid deployment in athletic training environments. This provides a replicable methodology and validation framework for future wearable and remote-monitoring technologies.

### 4.8. Sensor System Technical Specifications and Quality Assurance

All sensors met or exceeded manufacturer specifications and international calibration standards [20]: isokinetic dynamometer—monthly ISO 6954 calibration with precision weights, 100 Hz digital sampling, and 10 Hz low-pass Butterworth filter (cutoff 6–10 Hz depending on analysis phase) [10]; goniometer—bilateral inter-rater reliability ICC 0.94, intra-rater intra-day reliability ICC 0.97, standardized room temperature (22–24 °C), and standardized time of day (07:00–09:00 h) [33]; infrared thermometry—daily two-point calibration (25 °C and 45 °C reference blocks), ±2% accuracy verified across measurement range, and 1 Hz continuous sampling during thermal applications [18,29]; and ELISA—monthly plate-reader verification (wavelength filters), daily QC controls (low, medium, high concentrations), QC recovery rates 98.2–99.7%, intra/inter-assay CVs < 7%, and storage integrity verified via temperature logging (±2 °C freezer precision) [16,33].

### 4.9. Study Limitations, Generalizability, and Future Sensor Development

Despite high reliability and cross-modality concordance, several limitations warrant acknowledgment. First, this study enrolled exclusively young (mean age 21.88 ± 0.95 years), male, elite athletes with extensive training status (mean career 10.12 ± 1.44 years). This homogeneous cohort severely limits generalizability to broader populations. Critical sex-based differences remain poorly characterized: female athletes exhibit distinct hormonal profiles (estrogen, progesterone fluctuations), different muscle mass distribution patterns, altered body composition (adipose thickness), and substantially different inflammatory biomarker baseline values compared to male athletes. These physiological differences directly impact EIMD recovery kinetics and thermal intervention response; therefore, results cannot be extrapolated to female athletes without sex-specific validation studies. Furthermore, results do not generalize to older populations (>35 years), where age-related declines in satellite cell function, mitochondrial density, and protein synthesis capacity substantially compromise recovery capacity. Additionally, older adults exhibit altered inflammatory kinetics (elevated baseline pro-inflammatory cytokines, delayed IL-6 and CRP clearance), which fundamentally change the recovery phenotype and likely attenuate MWD intervention efficacy. Recreational or moderately trained athletes typically demonstrate different baseline strength levels, neuromuscular fatigue resistance, and metabolic reserve compared to elite athletes; therefore, the magnitude and timeline of recovery observed here may not transfer to less-trained populations. Finally, patients with pre-existing musculoskeletal conditions, chronic inflammatory states, or prior injury history likely exhibit altered inflammatory trajectories and compromised tissue regeneration that would fundamentally change both baseline recovery and intervention response. Future studies systematically incorporating female athletes, older adults (>40 years), recreational athletes, and clinical populations are essential to establish generalizability across diverse populations [17,36]. Second, infrared thermometry measures surface temperature; intramuscular thermal distribution was inferred from literature-derived calibration curves rather than direct invasive measurement [18]. While the consistency of thermal sensor response (coefficient of variation 14.3%) across all treated participants supports the validity of assumed deep-tissue heating, future studies incorporating fiber-optic intramuscular temperature probes could directly validate assumed 40–42 °C intramuscular temperatures and quantify thermal penetration gradients [29]. Such direct thermal sensing would enhance the thermal sensor subsystem’s mechanistic insight, though practical and ethical considerations regarding invasiveness may limit clinical deployment feasibility [29]. Third, the isokinetic dynamometer’s 10 Hz low-pass Butterworth filter necessarily attenuates torque fluctuations >5 Hz and eliminates signals >50 Hz. While this filtering strategy is standard for clinical peak torque quantification, it obscures potentially informative high-frequency neuromuscular phenomena. Specifically, motor unit synchronization patterns (20–40 Hz), muscle tremor characteristics (8–12 Hz), and rapid torque transients associated with neuromuscular fatigue or early regeneration can be attenuated or eliminated by this filter. These high-frequency signatures sometimes contain sensitive, early indicators of motor control adaptation or incomplete neuromuscular recovery that would be missed by conventional torque analysis. Alternative filtering approaches (e.g., 30–50 Hz cutoff, adaptive filters responsive to signal characteristics) could be explored in future studies to preserve high-frequency neuromuscular information while attenuating instrumental noise. However, the main effects and recovery trajectories in the present trial were robust, occurring at low frequencies (<5 Hz) and unlikely to be substantially influenced by filtering choices. Fourth, ELISA biomarker assessments (four timepoints across 72 h) represent discrete rather than continuous monitoring [16]. The temporal resolution is insufficient to capture peak inflammatory responses occurring between assessment windows or to detect rapid biomarker fluctuations. Future studies employing continuous biosensors (e.g., implantable or wearable systems) could improve temporal resolution and provide more granular inflammatory kinetics [15]. Additionally, ELISAs operate within defined detection ranges (CK 10–5000 U/L; IL-6 0.7–50 pg/mL; CRP 0.15–5.0 mg/L); samples exceeding upper limits require dilution, potentially introducing measurement variability [8,16]. In the present trial, all observed biomarker concentrations remained well within assay linearity ranges (IL-6 peak 15.93 pg/mL, within 0.7–50 pg/mL range), mitigating this concern. Fifth, the single thermal modality (skin surface) limits the spatial resolution of thermal distribution; future multi-channel thermal imaging or thermographic mapping could enhance the thermal sensor subsystem to capture three-dimensional thermal gradients and heterogeneous heating patterns [18]. Despite these technical considerations, the present study demonstrates the feasibility and clinical utility of integrated multi-sensor monitoring for comprehensive recovery assessment [15]. Future research should address population generalizability and employ direct intramuscular thermal validation to strengthen the mechanistic validity of the thermal monitoring subsystem [18,29].

### 4.10. Future Directions: Wearable Sensor Integration and Remote Monitoring

The success of the present fixed-laboratory, integrated multi-sensor platform provides a foundation for next-generation wearable and remote-monitoring technologies [34]. Emerging technologies such as miniaturized accelerometers and gyroscopes (integrated circuit MEMS sensors), wireless electrogoniometers, stretchable strain sensors for muscle deformation tracking, and point-of-care ELISA devices (microfluidic biosensors) could enable continuous, synchronized recovery monitoring in field-based athletic environments [15]. Cloud-based data fusion algorithms could integrate multimodal sensor streams, provide automated quality checks, flag abnormal recovery trajectories, and deliver timely coaching feedback [20]. Such sensor-driven decision-support systems may enhance training load management, optimize return-to-play timelines, and reduce re-injury risk in athletic populations [15]. The present study’s demonstration of multi-sensor concordance and cross-modality temporal synchronization provides a methodological template and validation framework for future wearable sensor development targeting comprehensive athletic health surveillance [20].

## 5. Conclusions

This study demonstrates the development, rigorous validation, and successful clinical application of a comprehensive multi-sensor integrated platform for objective, time-synchronized assessment of skeletal muscle recovery kinetics following acute exercise-induced muscle damage. The integrated platform, combining isokinetic dynamometry (biomechanical sensor, ICC 0.93, 100 Hz sampling, ±1% accuracy), goniometry (range-of-motion sensor, ICC 0.94–0.97, 1° resolution), infrared thermometry (thermal sensor, ±2% accuracy, real-time monitoring), and high-sensitivity ELISA (biochemical sensor array: CK, IL-6, CRP; quality-control recovery > 98%, intra/inter-assay CV < 7%), provided synchronized, multidimensional recovery phenotyping across biomechanical, thermal, and molecular domains. Pre-study validation established high reliability for all sensor channels, and cross-modality concordance analysis (isokinetic torque vs. IL-6, r = −0.73; pain vs. IL-6, r = 0.68; both *p* < 0.001) confirmed that recovery trajectories synchronize across distinct physiological systems.

Microwave diathermy (2.45 GHz, 160 W, 45 min/session at 24, 48, and 72 h intervals post-EIMD) intervention demonstrated efficacy quantifiable across all three sensor channels: substantial functional recovery (ROM and isokinetic torque restoration) and systemic inflammatory resolution (IL-6, CRP normalization) by 72 h in the treatment group, although serum CK remained above baseline, suggesting incomplete myofiber membrane integrity recovery in the treatment group, with persistent deficits in controls. The integrated sensor platform provided discriminative power and precision superior to any single-modality assessment, enabling comprehensive phenotyping of recovery, including temporal dissociation of functional versus inflammatory normalization phases.

The present study addresses critical methodological gaps identified in recent systematic reviews of sensor-based athletic monitoring, specifically the absence of comprehensive, multi-channel sensor systems for post-injury recovery assessment. By demonstrating the technical feasibility, analytical rigor, and clinical utility of integrated multi-sensor monitoring in a controlled trial setting, this work provides a foundation and methodological template for future wearable and remote-sensing technologies targeting continuous health surveillance in athletic populations. Further development toward portable, wireless, continuous sensor integration and cloud-based data fusion algorithms represents a promising direction for next-generation athletic health monitoring systems, with potential to enhance training load management, optimize return-to-play decision-making, and reduce re-injury risk in elite and recreational sports populations.

## Figures and Tables

**Table 1:** Physical characteristics of the participants.

Group (N = 40)	Age (yrs.)	Height (cm)	Weight (kg)	Career (yrs.)	DM
**MWDG (*n* = 20)**	21.88 ± 0.95	175.52 ± 1.64	87.80 ± 5.22	10.12 ± 1.44	Rt.
**CG (*n* = 20)**	21.19 ± 1.58	175.38 ± 1.27	84.98 ± 2.10	11.04 ± 1.89	

Values are mean ± standard deviation; MWDG: microwave diathermy group, CG: control group, DM: dominant, Rt: right.

**Table 2:** Microwave diathermy application protocol.

Trial	Type & Region	Intervention	Note
**EMDP**	Isokinetic ex. (KneeExt/Flx.60°/s) 20 × 10 set (set rest 60 s)	80% of MVC peak torque	Frequency 2.45 GHz Power 160 W
	Climb Stepmill 20 min × 3 (set rest 180 s)	80% of VO_2_max	
**MWD**	Quadriceps m.	15 min	
	Hamstring m.	15 min	
	Calf, AT, PT m.	15 min	
**Total time**		**45 min**	

EMDP: exercise muscle damage protocol; MWD: microwave diathermy; ex: exercise; Ext: extension; Flx: flexion.

**Table 3:** Changes in knee flexion range of motion (Unit: °).

Trial (N = 40)	Pre-Test (Baseline)	Post-1 (24 h)	Post-2 (48 h)	Post-3 (72 h)	Time		Group		Interaction		η^2^_p_
					F	P	F	P	F	P	
**MWDG**	130.14 ± 4.39 ^a^	120.16 ± 3.74 ^abc^	128.51 ± 4.10 ^b^	129.60 ± 3.53 ^c^	45.967	0.001 **	13.274	0.001 **	5.583	0.023 *	0.259 (Large)
**CG**	130.60 ± 4.71 ^def^	122.30 ± 4.05 ^d^	122.90 ± 4.16 ^e^	123.99 ± 4.69 ^f^							

Values are mean ± SD. MWDG: Microwave diathermy group; CG: control group; * *p* < 0.05, ** *p* < 0.001 by two-way repeated-measures ANOVA with Bonferroni post hoc correction unless otherwise specified. MWDG: ^a^ *p* < 0.001 (pre-test vs. post-1), ^b^ *p* < 0.001 (post-1 vs. post-2). CG: ^c^ *p* < 0.001 (pre-test vs. post-1), ^d^ *p* < 0.001 (pre-test vs. post-2), ^e^ *p* < 0.001 (pre-test vs. post-3), ^f^ *p* < 0.001 (pre-test vs. post-3. η^2^_p_: partial eta-squared.

**Table 4:** Changes in isokinetic peak torque at 60°/s (Unit: Nm).

Trial (N = 40)		Pre-Test (Baseline)	Post-1 (24 h)	Post-2 (48 h)	Post-3 (72 h)	Time		Group		Interaction		η^2^_p_
						F	P	F	P	F	P	
**Ex.**	**MWDG**	314.28 ± 21.43 ^ab^	164.20 ± 9.20 ^acd^	272.79 ± 9.08 ^bce^	310.72 ± 10.97 ^de^	802.063	0.001 **	345.308	0.001 **	201.786	0.001 **	0.841 (Large)
	**CG**	325.31 ± 15.06 ^fgh^	143.51 ± 14.22 ^f^	154.68 ± 16.63 ^gj^	183.31 ± 30.36 ^hij^							
**Fx.**	**MWDG**	187.20 ± 14.14 ^ab^	110.00 ± 9.00 ^acd^	159.76 ± 12.91 ^bce^	193.20 ± 14.37 ^de^	440.568	0.001 **	128.282	0.001 **	118.003	0.001 **	0.756 (Large)
	**CG**	206.40 ± 12.98 ^fgh^	99.77 ± 11.96 ^fij^	105.93 ± 14.99 ^gik^	123.98 ± 12.17 ^hjk^							

Values are mean ± SD. MWDG: Microwave diathermy group; CG: control group. ** *p* < 0.001 by two-way repeated-measures ANOVA with Bonferroni post hoc correction. Extension: ^a^ *p* < 0.001 (pre-test vs. post-1), ^b^ *p* < 0.001 (post-1 vs. post-2), ^c^ *p* > 0.05 (post-2 vs. post-3), ^d^ *p* < 0.001 (pre-test vs. post-1), ^e^ *p* < 0.001 (pre-test vs. post-2), ^f^ *p* < 0.001 (pre-test vs. post-3), ^g^ *p <* 0.001 (pre-test vs. post-1), ^h^ *p <* 0.001 (pre-test vs. post-2), ^i^ *p <* 0.001 (pre-test vs. post-3), ^j^ *p <* 0.001 (post-2 vs. post-3). Flexion: ^a^ *p* < 0.001 (pre-test vs. post-1), ^b^ *p* < 0.001 (post-1 vs. post-2), ^c^ *p* > 0.05 (post-2 vs. post-3). Flexion: ^d^ *p* < 0.001 (pre-test vs. post-1), ^e^ *p* < 0.001 (pre-test vs. post-2), ^f^ *p* < 0.001 (pre-test vs. post-3), ^g^ *p <* 0.001 (pre-test vs. post-1), ^h^ *p <* 0.001 (pre-test vs. post-2), ^i^ *p <* 0.001 (pre-test vs. post-3), ^j^ *p <* 0.001 (post-2 vs. post-3), ^k^ *p <* 0.001 (post-2 vs. post-3). η^2^_p_: partial eta-squared.

**Table 5:** Changes in VAS pain scores (Unit: mm).

Trial (N = 40)	Pre-Test (Baseline)	Post-1 (24 h)	Post-2 (48 h)	Post-3 (72 h)	Time		Group		Interaction		η^2^_p_
					F	P	F	P	F	P	
**MWDG**	0 ^abc^	59.88 ± 5.97 ^ade^	17.23 ± 3.41 ^bdf^	1.70 ± 2.50 ^cef^	1045.720	0.001 **	1884.124	0.001 **	353.255	0.001 **	0.903 (Large)
**CG**	0 ^ghi^	68.95 ± 7.05 ^g^	73.06 ± 8.74 ^hj^	62.95 ± 5.53 ^ij^							

Values are mean ± SD. MWDG: Microwave diathermy group; CG: control group; VAS: Visual Analogue Scale. ** *p* < 0.001 by two-way repeated-measures ANOVA with Bonferroni post hoc correction unless otherwise specified. MWDG: ^a^ *p* < 0.001 (pre-test vs. post-1), ^b^ *p* < 0.001 (post-1 vs. post-2), ^c^ *p* < 0.05 (pre-test vs. post-3). ^d^ *p* < 0.001 (pre-test vs. post-1), ^e^ *p* < 0.001 (pre-test vs. post-2), ^f^ *p* < 0.001 (post-2 vs. post-3), CG: ^g^ *p <* 0.001 (pre-test vs. post-1), ^h^ *p <* 0.001 (pre-test vs. post-2), ^i^ *p <* 0.001 (pre-test vs. post-3), ^j^ *p <* 0.001 (post-2 vs. post-3). η^2^_p_: partial eta-squared.

**Table 6:** Changes in serum creatine kinase (unit: U/L).

Trial (N = 40)	Pre-Test (Baseline)	Post-1 (24 h)	Post-2 (48 h)	Post-3 (72 h)	Time		Group		Interaction		η^2^_p_
					F	P	F	P	F	P	
**MWDG**	130.32 ± 11.32 ^abc^	212.32 ± 14.96 ^ade^	194.49 ± 17.02 ^bdf^	169.70 ± 22.58 ^cef^	186.018	0.001 **	262.035	0.001 **	38.848	0.001 **	0.506 (Large)
**CG**	128.74 ± 13.04 ^ghi^	315.24 ± 13.92 ^g^	317.75 ± 35.35 ^hj^	208.00 ± 60.74 ^ij^							

Values are mean ± SD. MWDG: Microwave diathermy group; CG: control group; All *p*-values < 0.001 by two-way repeated-measures ANOVA with Bonferroni post hoc correction unless otherwise specified. ** *p* < 0.001. ^a^ *p* < 0.001 (pre-test vs. post-1), ^b^ *p* < 0.05 (post-1 vs. post-2), ^c^ *p* < 0.001 (post-2 vs. post-3), ^d^ *p* < 0.001 (pre-test vs. post-1), ^e^ *p* < 0.001 (pre-test vs. post-2), ^f^ *p* < 0.001 (post-1 vs. post-3 and post-2 vs. post-3), ^g^ *p* < 0.001 (pre-test vs. post-1), ^h^ *p* < 0.001 (pre-test vs. post-2), ^i^ *p* < 0.001 (pre-test vs. post-3), ^j^ *p* < 0.001 (post-2 vs. post-3). η^2^p: Partial eta-squared.

**Table 7:** Changes in serum interleukin-6 (unit: pg/mL).

Trial (N = 40)	Pre-Test (Baseline)	Post-1 (24 h)	Post-2 (48 h)	Post-3 (72 h)	Time		Group		Interaction		η^2^_p_
					F	P	F	P	F	P	
**MWDG**	1.37 ± 0.13 ^ab^	15.93 ± 3.35 ^acd^	1.52 ± 0.14 ^bce^	1.35 ± 0.11 ^de^	294.813	0.001 **	261.278	0.001 **	65.002	0.001 **	0.631 (Large)
**CG**	1.46 ± 0.24 ^fgh^	19.57 ± 3.75 ^fi^	16.84 ± 5.01 ^gj^	9.32 ± 1.74 ^hij^							

Values are mean ± SD. MWDG: Microwave diathermy group; CG: control group; ** *p* < 0.001 by two-way repeated-measures ANOVA with Bonferroni post hoc correction unless otherwise specified. ^a^ *p* < 0.001 (pre-test vs. post-1), ^b^ *p* < 0.05 (post-1 vs. post-2), ^c^ *p* < 0.001 (post-2 vs. post-3), ^d^ *p* < 0.001 (pre-test vs. post-1), ^e^ *p* < 0.001 (pre-test vs. post-2), ^f^ *p* < 0.001 (post-1 vs. post-3 and post-2 vs. post-3), ^g^ *p* < 0.001 (pre-test vs. post-1), ^h^ *p* < 0.001 (pre-test vs. post-2), ^i^ *p* < 0.001 (pre-test vs. post-3), ^j^ *p* < 0.001 (post-2 vs. post-3). η^2^_p_: Partial eta-squared.

**Table 8:** Changes in serum C-reactive protein (unit: mg/L).

Trial (N = 40)	Pre-Test (Baseline)	Post-1 (24 h)	Post-2 (48 h)	Post-3 (72 h)	Time		Group		Interaction		η^2^_p_
					F	P	F	P	F	P	
**MWDG**	0.62 ± 0.25 ^abc^	5.37 ± 0.82 ^ade^	2.17 ± 0.25 ^bdf^	0.73 ± 0.24 ^cef^	613.471	0.001 **	403.967	0.001 **	124.186	0.001 **	0.766 (Large)
**CG**	0.67 ± 0.26 ^abc^	6.21 ± 0.86 ^ae^	5.85 ± 0.76 ^df^	4.60 ± 0.67 ^cef^							

Values are mean ± SD (n = 20/group). MWDG: Microwave diathermy group; CG: control group. ** *p* < 0.001 by two-way repeated-measures ANOVA with Bonferroni post hoc correction unless otherwise specified. ^a^ *p* < 0.001 (pre-test vs. post-1), ^b^ *p* < 0.001 (post-1 vs. post-2), ^c^ *p* > 0.05 (pre-test vs. post-3). ^d^ *p* < 0.001 (pre-test vs. post-1), ^e^ *p* < 0.001 (pre-test vs. post-2), ^f^ *p* < 0.001 (pre-test vs. post-3). η^2^p: Partial eta-squared.

## Data Availability

The original contributions presented in this study are included in the article. Further inquiries can be directed to the corresponding author.
